# Transcutaneous electrical nerve stimulation in speech therapy rehabilitation of voice and swallowing function in adults—a systematic review

**DOI:** 10.1002/cre2.470

**Published:** 2021-09-29

**Authors:** Milena Assis da Silva, Laura Davison Mangilli

**Affiliations:** ^1^ Rehabilitation Sciences Program Faculdade de Ceilândia, University of Brasília Brasília Brazil

**Keywords:** speech therapy, swallowing function, TENS, voice

## Abstract

**Introduction:**

In recent years, a number of clinical trials have been published comparing transcutaneous electrical nerve stimulation (TENS) and traditional speech therapy treatment of voice and swallowing functions, but results have been conflicting.

**Objective:**

Assess the methodological quality of studies and determine whether TENS is an efficient therapeutic strategy for speech therapy treatment of healthy adults or those with dysphonia and/or dysphagia.

**Methods:**

The databases used were Medical Literature Analysis and Retrieval System Online (MedLine), *Biblioteca Virtual em Saúde* (BVS), Cochrane Library and Web of Science (ISI Web of Knowledge). The study was conducted between May 2018 and January 2019, in line with Cochrane Handbook guidelines, and included studies on the use of TENS in healthy adults or those with compromised voice and/or swallowing function.

**Results:**

After the search and extraction of studies, the following were identified: TENS + VOICE: 7 articles; TENS + SWALLOWING: 5 articles. The studies exhibited medium quality and are heterogeneous, making it difficult to determine their effectiveness and the parameters to be used in future research. There were no statistically significant differences between the use of TENS alone or associated with another therapeutic technique for voice. For swallowing function, one study proved better results in cases of associated techniques – TENS + traditional therapy.

**Discussion:**

Speech therapy should increase the number of studies published and improve their methodological quality, reassessing methodological criteria. Current clinical practice is not grounded in evidence‐based science.

**Clinical Message:**

- the studies analyzed exhibited medium methodological quality;
- there are variations in the time, number and periodicity of the therapeutic sessions for TENS;
- there were no statistically significant differences between the use of TENS alone or associated with another therapeutic technique in voice;
- there were statistically significant differences between the use of TENS associated with traditional therapeutic in swallowing function.

## 1 INTRODUCTION

Transcutaneous electrical stimulation (TES) is currently being used by specialists to control pain, enhance muscle performance, stimulate wound healing, and improve sensorimotor recovery after different diseases (Blumenfeld et al., 2006; Guirro et al., 2008). It is a safe, noninvasive, simple, inexpensive, and nonpharmacological method (Mansuri et al., 2020; Santos et al., 2016). It seems that neuromotor function can be influenced by one TES parameter: amplitude (Barikroo et al., 2017; Doucet et al., 2012).

In the field of speech therapy, studies using TES remain scarce and, in some cases, are limited to the preliminary or initial therapy phase, where the technique is used to relax laryngeal muscles (Conde et al., 2017; Guirro et al., 2008; Silvério et al., 2015; Siqueira et al., 2017) or for the rehabilitation of mechanical oropharyngeal dysphagia, promoting muscle contraction (Crary & Carnaby, 2014). Several studies have been conducted in this area, showing the increasing use of new speech therapy resources (Guirro et al., 2008; Santos et al., 2016; Stangherlin et al., 2020). For a new therapeutic approach to be accepted, it should provide the same degree of benefits as proven techniques already in use. In recent years, a number of clinical trials have compared TES with traditional speech therapy for voice disorders (Conde et al., 2017; Fowler et al., 2011; Guirro et al., 2008; Silvério et al., 2015; Siqueira et al., 2017) and dysphagia (Blumenfeld et al., 2006; Crary & Carnaby, 2014; Maeda et al., 2017), but these articles reported conflicting results.

TES has been applied in clinical practice in two forms—motor and sensory electrostimulation. Motor electrostimulation or neuromuscular electrical stimulation (NMES) uses low‐intensity electrical currents to simulate the passage of a nervous stimulus to the skeletal muscle, promoting involuntary muscle contraction by depolarizing nerve fibers within the region of application (Bhatt et al., 2015; Crary & Carnaby, 2014; Glanz et al., 1996; Humbert et al., 2012; Simonelli et al., 2019). Sensory electrostimulation or transcutaneous electrical nerve stimulation (TENS) has been used to control pain and tension, reduce fatigue, improve local vascularity, help muscle relaxation and as analgesia in the region of application (Conde et al., 2017; Guirro et al., 2008; Mansuri et al., 2020; Santos et al., 2016; Silvério et al., 2015; Sluka & Walsh, 2003; Stangherlin et al., 2020). The two types of stimulus use percutaneous electrodes to transmit waveforms through the skin to stimulate large diameter nerve fibers (Mansuri et al., 2020; Santos et al., 2016; Silvério et al., 2015; Sluka & Walsh, 2003).

The use of low‐frequency TENS is related to symptomatic pain relief (acute or chronic), muscle relaxation (Conde et al., 2017; Guirro et al., 2008; Santos et al., 2016; Silvério et al., 2015) analgesic action (Guirro et al., 2008; Santos et al., 2016; Siqueira et al., 2017) and improved vascularization in the application region (Conde et al., 2017; Guirro et al., 2008; Santos et al., 2016; Silvério et al., 2015; Siqueira et al., 2017). The literature indicates that this technique stimulates the central inhibitory system (Conde et al., 2017; Guirro et al., 2008; Santos et al., 2016; Silvério et al., 2015; Siqueira et al., 2017). When used at low frequency (with intensity at the motor threshold), it stimulates nociceptive and efferent motor fibers, manipulates the output pattern of motoneuron activity and adds to the inherent activity of the muscles of the stimulated location (Guirro et al., 2008; Silvério et al., 2015). In patients with muscle tension, dysphonia studies suggest TENS as adjunct to voice therapy to decrease laryngeal symptoms and pain (Conde et al., 2017; Mansuri et al., 2020; Santos et al., 2016; Silvério et al., 2015).

Studies show benefits of TENS or NMES in improving swallowing functions (Barikroo et al., 2017; Barikroo et al., 2018; Berretin‐Felix et al., 2016; Blumenfeld et al., 2006; Carnaby & Harenberg, 2013), supporting hyolaryngeal elevation (Park et al., 2009; Park et al., 2012), reducing treatment sessions and shortening hospital stays (Blumenfeld et al., 2006). Other studies use TENS to increase oropharyngeal sensory input. This stimulus may increase sensory input to the swallowing center of the brain stem, leading to earlier initiation of deglutition and timely protection of the respiratory airway. Research demonstrates that periodical sensory stimulation may induce cortical neuroplasticity (Ortega et al., 2016; Rofes et al., 2014). As such, TES is still used for swallowing rehabilitation (Barikroo et al., 2017; Carnaby & Harenberg, 2013), and is the most widely studied approach to dysphagia rehabilitation (Crary & Carnaby, 2014). However, there is a lack of evidence in the literature (Crary & Carnaby, 2014), highlighting the relatively weak research designs, small studies, and the use of different electrical stimulation parameters for dysphagic patients (e.g., stroke, older healthy adults, progressive diseases, head/neck cancers).

Given that TENS is a new important therapeutic strategy for voice and swallowing functions, a systematic review was conducted to analyze current evidence on TENS obtained in experimental and quasi‐experimental studies with healthy, dysphonic and dysphagic adults without other pathologies (e.g., neurological or head and neck cancer). The information in this review may help establish and develop rehabilitation programs aimed at adults with dysphonia and/or dysphagia.

## 2 METHODS

This systematic review was conducted according to the recommendations of the Cochrane Handbook (Higgins et al., 2019). All the stages described were carried out by two independent examiners, with disagreements resolved by a third examiner.

The following databases were surveyed between May 2018 and January 2019: *Biblioteca Virtual em Saúde* (BVS), Medical Literature Analysis and Retrieval System Online (PubMed/MedLine), Cochrane Library and Web of Science (ISI Web of Knowledge), with no restriction for language or year of publication.

The search for articles in the areas of voice and swallowing function and their association with TENS was performed in three stages: the first involved two searches for articles in the databases. This search was conducted separately, considering the voice and swallowing areas; the second stage excluded duplicate articles, namely those contained in more than one database, or appearing as different documents in the same research; and the third involved reading and analyzing the texts considering the established inclusion criteria.

The research strategy used for the BVS applied the following descriptors: 1—“transcutaneous electrical nerve stimulation” OR “sensory electrical stimulation” OR “sensory e‐stim” OR “sensory transcutaneous electrical stimulation” OR “sensory TES”) AND (voz OR voice OR disfonia OR “*distúrbios da voz*”) AND *adultos*; 2—“transcutaneous electrical nerve stimulation” OR “sensory electrical stimulation” OR “sensory e‐stim” OR “sensory transcutaneous electrical stimulation” OR “sensory TES”) AND (*deglutição* OR “*distúrbios da deglutição*”) AND *adultos*.

The following descriptors were used on the PUBMED and Cochrane Library platforms: 1—(“transcutaneous electric nerve stimulation” OR “sensory electrical stimulation” OR “sensory e‐stim” OR “sensory transcutaneous electrical stimulation” OR “sensory TES”) AND (voice OR dysphonia OR “voice disorders”) AND adults; 2—(“transcutaneous electric nerve stimulation” OR “sensory electrical stimulation” OR “sensory e‐stim” OR “sensory transcutaneous electrical stimulation” OR “sensory TES”) AND (deglutition OR “deglutition disorders”) AND adults.

The descriptors used on the Web of Science (ISI Web of Knowledge) database were: 1—(“transcutaneous electric stimulation” OR “transcutaneous electrical stimulation” OR “trans‐cutaneous electrical nerve‐stimulation” OR “transcutaneous nerve stimulation” OR “transcutaneous electrical nerve stimulation” OR “sensory electrical stimulation” OR “sensory e‐stim” OR “sensory transcutaneous electrical stimulation” OR “sensory TES”) AND (“voice OR dysphonia” OR “voice disorders”) AND adults; 2—(“transcutaneous electric stimulation” OR “transcutaneous electrical stimulation” OR “trans‐cutaneous electrical nerve‐stimulation” OR “transcutaneous nerve stimulation” OR “transcutaneous electrical nerve stimulation” OR “sensory electrical stimulation” OR “sensory e‐stim” OR “sensory transcutaneous electrical stimulation” OR “sensory TES”) AND (deglutition OR “deglutition disorders”) AND adults.

### 2.1 Study selection

The following article eligibility criteria were established (according to the PICO tool): (1) healthy (cases of vocal or deglutition improvement), dysphonic or dysphagic adult sample (neurological, psychiatric, syndromic and diagnosed head and neck cancer cases were excluded to reduce bias); (2) use of TENS as an intervention method; (3) pre and post‐intervention comparison; (4) at least one of the following voice or swallowing parameters: results of a specific clinical evaluation (e.g., vocal quality scale or swallowing scale); self‐perception scale/instruments; diadochokinetic performance; acoustic analysis; face and neck muscle activity; and videofluoroscopy; and (5) published reports of experimental and quasi‐experimental studies.

The articles were selected independently by two examiners. The titles were read and those that did not meet eligibility criteria were excluded. Next, the abstracts were read and the studies of those that were not excluded were read in their entirety to select the articles included in this review.

### 2.2 Data extraction and quality assessment

The material was critically assessed considering the methodological quality and the risk of bias of the selected articles, using a standardized scale (Higgins et al., 2019; Sampaio & Mancini, 2017; Shiwa et al., 2011). To understand TENS application for voice and swallowing function, a critical review of the studies with high‐quality methodology was carried out, analyzing the objectives, results and methodological standardization used.

The PEDro scale was developed by the Physiotherapy Evidence Database (Sampaio & Mancini, 2017; Shiwa et al., 2011) for use in experimental studies. The maximum score is 10 points and includes assessment criteria of internal validity and presentation of the statistical analysis used. The first item, related to the inclusion criteria, is considered positive or negative, instead of being scored. For the remaining criteria, 1 point is attributed when quality indicators are present and 0 points when they are absent. The scale is composed of the following criteria: (1) eligibility criteria specified (item not scored); (2) random allocation; (3) concealed allocation; (4) groups similar at baseline; (5) subject blinding; (6) therapist blinding; (7) assessor blinding; (8) less than 15% dropouts (measure of at least one primary outcome in 85% of the allocated subjects); (9) intention‐to‐treat analysis; (10) between‐group statistical comparisons (intergroup comparison of at least one primary outcome) (11) point measures and variability data (report of variability measures and parameter estimation of at least one primary variable) (Shiwa et al., 2011).

The scale was used independently and blindly by two researchers, and no disagreements occurred. After the final score was calculated, the following decisions were made (Sampaio & Mancini, 2017; Shiwa et al., 2011): (a) articles with scores of less than 3 were deemed to have low methodological quality and were excluded; (b) articles with scores greater than or equal to 3 were considered eligible for the next stage.

Following the recommendations of the Cochrane Handbook (Higgins et al., 2019), the risk of bias analysis was also applied to the studies selected for this review. The Cochrane tool provides a framework for assessing the risk of bias in a single result from any type of randomized trial. It is structured into domains, that will be identify based on both empirical evidence and theoretical considerations. The tool was also applied independently and blindly by two researchers, and no disagreements occurred.

The following variables were considered to analyze the use of TENS for voice and swallowing functions: study objective, participants, evaluations methods, TES application method (frequency, intensity, duration, and electrode location) and outcomes.

## 3 RESULTS

### 3.1 Study selection

After the search and extraction of studies, the following were identified: (1) TENS + VOICE: 35 studies, 20 of which were excluded for being duplicates. Seven of the 15 remaining articles met the eligibility criteria. Studies that were not experimental (systematic review or case reports) and those involving other diseases (two neurological, one chest and one digestive) were excluded. (2) TENS + SWALLOWING: 37 studies, 13 of which were excluded for being duplicates. Only 5 of the remaining articles met the eligibility criteria. Nine of the excluded studies involved neurologic patients, six were not experimental and four involved other disorders (three digestive and one pain‐related).

**FIGURE 1:**
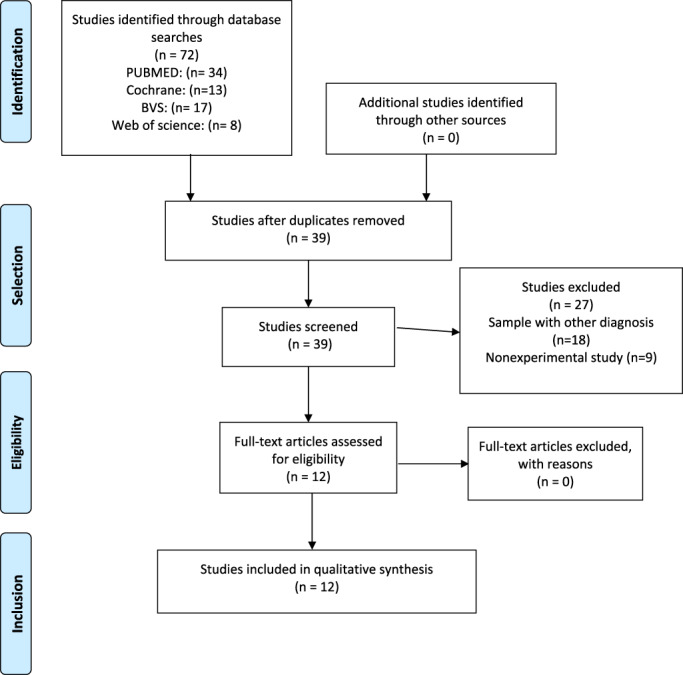
The flowchart of the review studies

The flowchart of the review studies is described in Figure 1.

### 3.2 Data extraction and quality assessment

Analysis of methodological quality did not exclude any of the articles selected. Table 1 presents the PEDro scale score of the articles. Table 2 shows the results of applying the Cochrane tool.

**TABLE 1:** Methodological quality assessment – PEDro scale

PEDro items	Voice							Swallowing				
	Conde et al. (2017)	Fabron et al. (2017)	Siqueira et al. (2017)	Santos et al. (2016)	Silvério et al. (2015)	Fowler et al. (2011)	Santos et al. (2016)	Maeda et al. (2017)	Ortega et al. (2016)	Barikroo et al. (2017)	Berretin‐Felix et al. (2016)	Blumenfeld et al. (2006)
1. Eligibility criteria	YES	YES	YES	YES	YES	YES	YES	YES	YES	YES	YES	YES
2. Randomized Allocation	YES	NO	YES	YES	YES	NO	NO	YES	YES	YES	YES	NO
3. Secret Allocation	YES	YES	NO	YES	NO	NO	NO	YES	YES	NO	NO	NO
4. Similarity of groups in prognosis	YES	YES	YES	YES	YES	YES	YES	YES	YES	YES	YES	YES
5. Blinding of all subjects	NO	NO	NO	YES	NO	NO	NO	YES	NO	YES	YES	NO
6. Blinding of all therapists	NO	NO	NO	NO	NO	NO	NO	NO	NO	NO	NO	NO
7. Blinding of assessor	NO	NO	NO	YES	NO	NO	NO	NO	NO	NO	NO	NO
8. Measures of at least one key outcome were obtained from more than 85% of the subjects initially allocated to groups	YES	YES	YES	YES	YES	YES	YES	YES	YES	YES	YES	YES
9. Analysis of “intention to treat”	YES	YES	YES	YES	YES	YES	YES	YES	YES	YES	YES	YES
10. Between‐group statistical comparisons	YES	YES	YES	YES	YES	0	0	YES	YES	YES	YES	YES
11. Precision and variability measures	YES	YES	YES	YES	YES	YES	YES	YES	YES	YES	YES	YES
Total point	7/10	6/10	6/10	9/10	6/10	4/10	4/10	8/10	7/10	7/10	7/10	5/10

**TABLE 2:** The risk of bias analysis – Cochrane tool

Risk of bias	Voice							Swallowing				
	Conde et al. (2017)	Fabron et al. (2017)	Siqueira et al. (2017)	Santos et al. (2016)	Silvério et al. (2015)	Fowler et al. (2011)	Santos et al. (2016)	Maeda et al. (2017)	Ortega et al. (2016)	Barikroo et al. (2017)	Berretin‐Felix et al. (2016)	Blumenfeld et al. (2006)
Random sequence generation	Low	Low	Low	Low	Low	High	High	Low	Low	Low	Low	High
Allocation concealment	Unclear	Unclear	Low	Low	Low	High	High	Low	Low	Low	Low	High
Blinding of patients, personnel	High	High	High	High	High	High	High	Low	Unclear	Unclear	Unclear	High
Blinding of outcome assessors	Low	Low	Low	Low	Low	High	High	Low	Unclaer	Unclear	Unclear	Unclear
Incomplete outcome data	Low	Low	Low	Low	Low	Low	Low	Low	Low	Low	Low	Low
Selective outcome reporting	Low	Low	Low	Low	Low	Low	Low	Low	Low	Low	Low	Low
Other												

*Note*: Low, low risk of bias; Unclear, unclear risk of bias; High, high risk of bias.

The summary of the variables analyzed in the articles is presented in Chart 1.

**CHART 1:** Summary of the variables analyzed in the articles—study characteristics

Data general	TES application						Outcome
Citation	Objective		Frequency	Intensity	Duration	Electrode location	
**Voice**							
Fabron et al. (2017)	Determine the variation in vocal quality after application of the VTVT associated with TENS of the larynx in women with normal laryngeal function	Healthy women	10 Hz	At the comfort threshold	A single 5‐minute session	3.5 x 4.5 cm^2^. Placed laterally in the larynx, on the thyroid cartilage	The results were not statistically significant, but suggest that the association between TENS and the VTVT technique influenced the feeling of discomfort during vocal emission and vocal quality
Siqueira et al. (2017)	Determine and compare the effects of LMT and TENS on laryngeal diadochokinesis in dysphonic women	Women with vocal fold nodules	10 Hz	At the motor threshold	A total of twelve 20‐minute sessions; twice a week	3.0 x 4.0 cm^2^. Placed in the trapezius muscle region and bilaterally in the submandibular region	The results were not statistically significant. It was determined that the TML and TENS provide muscle relaxation and can may be used together or alone to treat dysphonia. The group that received TML showed more regular diadochokinetic movements in the vocal folds. The group that received TENS showed no change in diadochokinetic parameters
Conde et al. (2017)	Determine the immediate effect of low‐frequency TENS and LMT on musculoskeletal pain, vocal quality, and self‐perception in women with dysphonia	Women with dysphonia	10 Hz	At the motor threshold	A single 20‐minute session	5.0 × 4.0 cm^2^. Placed on the motor point of the trapezius muscle and bilaterally in the submandibular region	The results were not statistically significant. There was no intergroup difference in the acoustic parameters. A single TENS or LMT session immediately improved vocal quality. The group that received TENS reported a more positive change in vocal quality than the group that received only LMT
Santos et al. (2016)	Assess the effect of TENS with and without the VVT	Women with vocal fold nodules	10 Hz	At the motor threshold	A single 20‐minute session	30 x 50 mm^2^, bilaterally on the thyroid cartilage, and bilaterally on the trapezius muscle	The results were not statistically significant, but the group that received TENS associated with the voiced tongue vibration showed positive changes in the auditory parameters, phonatory exertion and self‐rating. The group that received only TENS improved glottic closure and phonatory comfort
Silvério et al. (2015)	Determine the effect of TENS and TML, and compare the two techniques in terms of vocal/laryngeal symptoms: pain and vocal quality after application in dysphonic women	Women with vocal fold nodules	10 Hz	At the motor threshold	A total of twelve 20‐minute sessions, twice a week	5.0 x 5.0 cm^2^. Bilaterally in the trapezius region and bilaterally in the submandibular region	The acoustic parameters showed no significant change after the two treatments. The group that received TENS showed a decline in vocal/laryngeal symptoms in terms of the frequency and intensity of musculoskeletal pain in the neck and shoulders, as well as improved vocal quality. The group that received LMT exhibited a reduction in throat and posterior neck pain, but no improvement in vocal quality
Fowler et al. (2011)	Determine whether there were measurable changes in fundamental frequency (f0) and RSL in healthy speakers after TENS RSL	Healthy adults	80 Hz	At the motor threshold	A single 1‐hour session	2.1 cm diameter and 3.46 cm^2^. Placed laterally to the midline of the submental region and on the sides of the cricothyroid region	No statistically significant data were found when comparing f0 and RSL before and after the use of TENS. There was considerable variation among the participants
Santos et al. (2016)	Bilaterally assess the electrical activity of suprahyoid, sternocleidomastoid and trapezius muscles, pain, and voice, after TENS	Women with dysphonia	10 Hz	At the motor threshold	A total of ten 30‐minute sessions, two or three times a week	4.0 × 4.0 cm^2^. Placed on the trapezius muscle and bilaterally on the sternocleidomastoids.	The average electromyographic activity values of the muscles analyzed decreased, except the right sternocleidomastoid in the emission of the E vowel and the suprahyoid in spontaneous speech. There was no difference in the pre‐ and post‐intervention acoustic parameters. Perceptive‐auditory analysis of vocal quality after TENS showed a significant decline in dysphonia
**SWALLOWING**							
**Studies that used only sensory electrostimulation/transcutaneous electrical nerve stimulation (TENS)**							
Blumenfeld et al. (2006)	Assess the effectiveness of ES in the treatment of patients with dysphagia and aspiration	Adults with dysphagia	80 Hz	At the motor threshold	30‐minute sessions. The number of sessions depended on the progress and treatment plan of each patient.	Electrodes placed horizontally just above the thyroid notch	Both groups (one received traditional dysphagia therapy and the other ES) showed a significant improvement on the dysphagia severity rating scale after the treatments. The ES group needed fewer treatment sessions and showed a trend towards less hospitalization time
Maeda et al. (2017)	Investigate the effect of SS, using interferential current, in patients submitted to dysphagia rehabilitation	Patients who received dysphagia rehabilitation in the hospital for 3 weeks	Two different alternating currents (2000 and 2050 Hz) are carried between pairs of electrodes, generating a 50‐beat interferential current	3.0 mA (insufficient to produce muscle contractions)	Twice‐a‐day 15‐minute sessions (morning and afternoon), 5 days a week, for 2 weeks	Electrodes placed on the anterior part at the border of the thyroid cartilage and posterior electrodes 4.0 cm from the ipsilateral electrode, along the mandible	There were no significant results, but the group that received SS using interferential current obtained better impacts on sensitivity to coughing and nutritional status among patients with dysphagia that did not receive SS
Ortega et al. (2016)	Assess and compare the effect of two long‐term sensory treatment strategies (TRPV and TSES)—in older patients with oropharyngeal dysphagia	Older patients with oropharyngeal dysphagia	80 Hz	Intensity of 75% of previously established motor threshold	A total of ten 1‐hour sessions, 5 days a week (Monday to Friday), for 2 weeks	Electrodes placed on the thyroid cartilage	The group that received TRPV agonists showed a significant decline of more than 2 points in the EAT‐10 score. The group that received TSES showed no statistical differences; both sensory strategies induced improvement in the prevalence of VFS signs and swallowing in older patients with oropharyngeal dysphagia
**Studies that used the both stimuli—sensory and motor electrostimulation/NMES and TENS**							
Barikroo et al. (2017)	Compare the effect of TES amplitude on timing of lingual–palatal and pharyngeal peak pressures during swallowing in healthy younger and older adults	Healthy young and older adults	80 Hz with pulse duration of 700 μs	Low‐amplitude stimulation was defined as 2 mA below the initial motor response amplitude, and high‐amplitude stimulation as 2 mA below the maximum tolerance amplitude	During the task—10 ml of nectar‐thick liquid under three TES conditions: no stimulation, low‐amplitude stimulation, and high‐amplitude stimulation	Electrodes placed on the suprahyoid and infrahyoid muscle groups	A significant age × stimulation amplitude interaction was identified for the base of the tongue and the hypopharynx. At the base of the tongue, low‐amplitude TES resulted in slower swallows in the younger adults compared with no TES. In older adults, low‐amplitude TES resulted in faster swallows compared with high‐amplitude TES. At the hypopharynx, no significant differences were identified in pressure timing across the three TES amplitudes in both age groups. In each case, low‐amplitude TES resulted in faster swallows in older than younger adults
Berretin‐Felix et al. (2016)	Compare the immediate impact of different TES amplitudes (compatible with sensory and motor stimulation) on physiological swallowing effort in healthy older adults versus young adults	Healthy young and older adults	80 Hz with pulse duration of 700 μs	SS was operationally defined as 2 mA below the initial motor response amplitude, and motor stimulation as 2 mA below the maximum tolerance amplitude.	During the tasks—a total of 27 swallows were evaluated for each participant (involving 3 stimulation conditions—motor, sensory or sham, 3 consistencies and 3 volumes)	Electrodes placed on the suprahyoid and infrahyoid muscle groups	There were interactions between age and stimulation amplitude on lingual and pharyngeal functions. Motor stimulation reduced anterior tongue pressure in both age groups but selectively reduced posterior lingual‐palatal pressures in young adults only. SS increased base of tongue pressures in older adults but decreased in young adults. Motor stimulation increased hypopharyngeal pressures in both groups

Abbreviations: %, percentage; cm, centimeters; EAT‐10, Eating Assessment Tool; ES, electrical stimulation; f0, fundamental frequency; Hz, hertz; LMT, Laryngeal Manual Therapy; mA, milliamps; ml, milliliters; mm, millimeters; NMES, neuromuscular electrical stimulation; RSL, relative sound level; SS, sensory stimulation; TENS, transcutaneous electrical nerve stimulation; TES, transcutaneous electrical stimulations; TRPV, transient receptor potential vanilloid; TSES, transcutaneous sensory electrical stimulation; VFS, videofluoroscopy; VTVT, voiced tongue vibration technique; μs, microseconds.

All the voice studies (Conde et al., 2017; Fabron et al., 2017; Fowler et al., 2011; Guirro et al., 2008; Santos et al., 2016; Silvério et al., 2015; Siqueira et al., 2017) used only sensory stimulation, and four investigated the effect of TENS on voice considering otolaryngologist assessment to determine vocal lesions. All the studies (Conde et al., 2017; Fabron et al., 2017; Fowler et al., 2011; Guirro et al., 2008; Santos et al., 2016; Silvério et al., 2015; Siqueira et al., 2017) made audio recordings of the participants, with perceptive‐auditory assessment by qualified professionals. In addition, for tension/pain, one study applied the Musculoskeletal Pain Questionnaire (Conde et al., 2017) and another the Nordic Musculoskeletal Symptoms Questionnaire (Silvério et al., 2015). For voice performance, one study (Fabron et al., 2017) also developed a self‐assessment protocol where participants rated their perception on a 10‐cm visual analogue scale. Another study (Santos et al., 2016) asked subjects if they perceived a change in their voice after the intervention. Some researchers (Siqueira et al., 2017) used audio recordings to assess only the diadochokinesis of participants and the data were analyzed by a computer program. We also highlight another study (Guirro et al., 2008) that used the Grade, Roughness, Breathiness, Asthenia, Strain, Instability scale to analyze voice parameters and the Superficial Electromyographic assessment to determine the electrical activity of the suprahyoid, sternocleidomastoid and trapezius muscle.

In some articles acoustic analysis was conducted (Fabron et al., 2017; Fowler et al., 2011; Guirro et al., 2008; Santos et al., 2016), considering fundamental frequency (f0) (Fabron et al., 2017; Fowler et al., 2011; Santos et al., 2016), jitter (Fabron et al., 2017; Guirro et al., 2008; Santos et al., 2016), shimmer (Fabron et al., 2017; Guirro et al., 2008; Santos et al., 2016), noise‐to‐harmonic ratio (Fabron et al., 2017; Guirro et al., 2008), and relative sound level (Fowler et al., 2011).

For swallowing function, three studies used only sensory stimulation (Blumenfeld et al., 2006; Maeda et al., 2017; Ortega et al., 2016) and two used both sensory and motor stimuli (Barikroo et al., 2017; Berretin‐Felix et al., 2016). The studies (Barikroo et al., 2017; Berretin‐Felix et al., 2016; Blumenfeld et al., 2006; Maeda et al., 2017; Ortega et al., 2016) used multidisciplinary assessment and/or the gold standard swallow test (Videofluoroscopy) to determine whether the participants had dysphagia. Different assessment instruments were used: cough latency time, the Functional Oral Intake Scale and Mini‐Nutritional Assessment Short Form in one study (Maeda et al., 2017); swallowing pressure data (lingual‐palatal and pharyngeal pressures) and pressure timing in two studies (Barikroo et al., 2017; Berretin‐Felix et al., 2016); the Swallow Severity Scale in one study (Blumenfeld et al., 2006); and the Eating Assessment Tool, Volume‐Viscosity Swallow Test and Penetration‐Aspiration Scale (for videofluoroscopy exam) in another (Ortega et al., 2016).

In relation to equipment, for voice, the most widely used device in studies was the Dualpex 961 (Quark Medical) (Conde et al., 2017; Guirro et al., 2008; Silvério et al., 2015; Siqueira et al., 2017), followed by Neurodyn II (IBRAMED) (Fabron et al., 2017; Santos et al., 2016) and the Vital Stim (DJO Global) (Fowler et al., 2011). For swallow function studies, the most widely used device for stimulation was the Vital Stim (DJO Global) (Barikroo et al., 2017; Berretin‐Felix et al., 2016; Blumenfeld et al., 2006; Ortega et al., 2016), followed by the portable Gentle Stim (J Craft) (Maeda et al., 2017).

With regard to frequency, most of the voice studies (Conde et al., 2017; Fabron et al., 2017; Guirro et al., 2008; Santos et al., 2016; Silvério et al., 2015; Siqueira et al., 2017) used a frequency of 10 Hz and only one (Fowler et al., 2011) an 80 Hz frequency. For swallowing functions, stimuli frequency differed. One study (Maeda et al., 2017) used interferential current sensory stimulation, with two different alternating currents, generating a 50‐beat interferential current, while four studies (Barikroo et al., 2017; Berretin‐Felix et al., 2016; Blumenfeld et al., 2006; Ortega et al., 2016) used the 80 Hz frequency.

With respect to intensity/amplitude, eight of the 12 studies considered the motor intensity threshold of each participant (Blumenfeld et al., 2006; Conde et al., 2017; Fabron et al., 2017; Fowler et al., 2011; Guirro et al., 2008; Santos et al., 2016; Silvério et al., 2015; Siqueira et al., 2017). The motor threshold was determined when a motor response was visually or verbally identified. Two studies (Barikroo et al., 2017; Berretin‐Felix et al., 2016) used low‐amplitude stimulation, (defined as 2 mA below the initial motor response) and high‐amplitude stimulation (defined as 2 mA below the maximum tolerance amplitude). Another study considered 75% of the motor threshold (Ortega et al., 2016) and, finally, the last (Maeda et al., 2017) used an intensity of 3.0 mA.

Stimulation duration, and the number and periodicity of the sessions differed between studies. In relation to duration, two studies (Blumenfeld et al., 2006; Guirro et al., 2008) used 30 minutes of continuous stimulation, four (Conde et al., 2017; Santos et al., 2016; Silvério et al., 2015; Siqueira et al., 2017) 20 minutes, two (Fowler et al., 2011; Ortega et al., 2016) stimulated individuals for 1 hour, one (Maeda et al., 2017) for 15 minutes, one (Fabron et al., 2017) for 5 minutes, and two (Barikroo et al., 2017; Berretin‐Felix et al., 2016) during the tasks.

One study (Blumenfeld et al., 2006) did not specify the number of sessions the individuals underwent, or how many times a day/week. The authors did not report the number of daily interventions or the total average, only that individuals were stimulated until they achieved the goal of the therapeutic treatment plan. One study (Maeda et al., 2017) conducted the largest number of sessions (20), twice a day for five consecutive days, over a two‐week period. Two studies (Silvério et al., 2015; Siqueira et al., 2017) held a total of 12 sessions, twice a week for 6 weeks. The other two studies used 10 sessions, 5 days a week for 2 weeks (Ortega et al., 2016) and two or three times a week (Guirro et al., 2008). The greatest number of articles (Barikroo et al., 2017; Berretin‐Felix et al., 2016; Conde et al., 2017; Fabron et al., 2017; Fowler et al., 2011; Santos et al., 2016) held only a single stimulation session.

Other aspects that varied between studies were the size and location of the electrodes used. With respect to electrode placement, six studies (Blumenfeld et al., 2006; Fabron et al., 2017; Fowler et al., 2011; Maeda et al., 2017; Ortega et al., 2016; Santos et al., 2016) placed electrodes on the thyroid cartilage. Five articles (Conde et al., 2017; Guirro et al., 2008; Santos et al., 2016; Silvério et al., 2015; Siqueira et al., 2017) placed electrodes on the trapezius muscle; four (Conde et al., 2017; Fowler et al., 2011; Silvério et al., 2015; Siqueira et al., 2017) considered the submandibular region; two (Barikroo et al., 2017; Berretin‐Felix et al., 2016) on the suprahyoid and infrahyoid muscle groups; and only one (Guirro et al., 2008) placed electrodes on the sternocleidomastoid muscle.

## 4 DISCUSSION

This systematic review studied 12 articles with methodology quality to analyze the current evidence on TENS application as a strategy in the rehabilitation/habilitation of voice and swallowing function.

In relation to the quality of the articles, according to the PEDro scale, none were excluded, that is, all obtained scores above the established threshold and specified the eligibility criteria used. The articles scored lower in the criteria related to the randomization and blinding of the individuals. Studies that did not randomize displayed bias in subject selection, which may interfere in group comparison. The studies that did not use participant blinding exhibited performance bias, but in those involving electrostimulation, complete blinding can be precluded (Higgins et al., 2019; Sampaio & Mancini, 2017; Shiwa et al., 2011).

There is significant heterogeneity in the studies, even when considering voice and swallowing function individually. Of the 12 articles analyzed, only the results of 2–3 could be compared with one another, precluding meta‐analysis to obtain the combined effect of a treatment, since a larger number of homogeneous studies are required.

Skeletal muscle power can be increased using nearly any strategy, provided exercise frequency and load intensity sufficiently exceed the normal or current activation of this muscle (Komi, 1986). Electrical stimulation can manipulate the output pattern of motoneuron activity and combines with muscle activity in the stimulated region, when compared to stimulation using voluntary exercises that gradually and hierarchically activate individual motorneurons (Kitchen, 2001). Furthermore, TENS stimulates the nerve and motor fibers. This activation triggers the descending analgesic systems of an inhibitory character in nociceptive transmission, thereby reducing pain (Santos et al., 2016; Silvério et al., 2015).

In recent years, studies have been carried out to investigate the use of TENS in dysphonic patients (Conde et al., 2017; Fabron et al., 2017; Fowler et al., 2011; Guirro et al., 2008; Santos et al., 2016; Silvério et al., 2015; Siqueira et al., 2017). Musculoskeletal pain reduction and improvements in vocal quality have been reported as the main objectives. This revision show that these studies report positive results in the use of stimulation, but when compared to other traditional therapies, the results did not show greater benefits.

In relation to the results obtained in the voice studies, of the three (Conde et al., 2017; Silvério et al., 2015; Siqueira et al., 2017) that compared the use of TENS with laryngeal manual therapy (LMT), only one (Siqueira et al., 2017), whose main variable was laryngeal diadochokinesis, demonstrated that the use of TENS did not show greater vocal fold movement regularity. The LMT would be a more suitable strategy to treat the diadochokinetic movements of the vocal folds. Another study (Fowler et al., 2011), which aimed to determine the immediate effect of TENS on the voice of healthy individuals, found positive changes in the voice, primarily in fundamental frequency, with no statistical difference.

The three studies (Conde et al., 2017; Guirro et al., 2008; Silvério et al., 2015) that used vocal quality and musculoskeletal pain to analyze the effect of TENS therapy reported favorable but not statistically significant results, when compared to the traditional therapy used. Despite not finding statistically significant differences, the two studies (Fabron et al., 2017; Santos et al., 2016) that compared the use of TENS alone and in conjunction with voiced tongue vibration obtained positive vocal quality results for both alternatives.

Recent studies suggested that the use of low‐frequency TENS (>10 Hz) in dysphonic patients, associated or not with voice therapy techniques, can help reduce pain and symptoms. Although they indicate a reduction in vocal symptoms, decline in pain and improved vocal quality after TENS stimulation, these studies are small in number, with few participants and present a medium level of evidence. A rigorous assessment of methodological quality is necessary to ensure that findings are more robust and replicable in clinical practice (Stangherlin et al., 2020).

For swallowing functions, TES is also applied as a direct intervention or an adjuvant treatment to exercise or to thermal‐tactile stimulation approaches (Crary & Carnaby, 2014; Lim et al., 2009; Ryu et al., 2009; Simonelli et al., 2019; Sun et al., 2013). Most studies agree that better results are achieved when TES is used as an adjuvant to traditional therapy (Carnaby‐Mann & Crary, 2007; Carnaby‐Mann & Crary, 2008; Lim et al., 2009; Ryu et al., 2009; Sun et al., 2013). For post‐stroke dysphagia patients, a meta‐analysis (Chen et al., 2015) showed that both treatments – traditional and NMES – were more effective in the short term. However, this review indicates insufficient evidence due to the limited number of studies conducted involving individuals with impaired swallowing without other disorders.

With respect to studies that focused on swallowing function in this review, only one (Blumenfeld et al., 2006) compared the use of TENS with traditional speech therapy, demonstrating a significant improvement for both treatment groups, according to the dysphagia classification scale. However, the results were more significant for individuals who received associated treatments. Another study (Maeda et al., 2017) also used the dysphagia classification scale to compare the stimulation and sham groups and found an improvement in dysphagia in individuals who received electrostimulation, albeit not significant. Another study (Ortega et al., 2016) compared two therapeutic strategies for sensory stimulation—TENS versus a natural solution—and observed similar intergroup parameter results, with no statistically significant difference. Some variables, such as hyoid muscle mobility, showed no change in either group, and the only difference the authors underscored was that the group that did not receive TENS obtained a lower score on the dysphagia risk scale at the end of therapy, a positive result for dysphagia rehabilitation.

The literature shows interactions between age and stimulation amplitude in tongue and pharyngeal pressure (Barikroo et al., 2017; Berretin‐Felix et al., 2016). In healthy adults, the studies demonstrated different TES amplitude effects (low vs. high) between younger and older adults: high amplitude reduced anterior lingual‐palatal pressure peaks, but increased hypopharyngeal pressure peaks for both age groups; and low amplitude increased pressure peaks in older adults, while reducing them in younger adults.

As described in this review, conflicting outcomes regarding the clinical effectiveness of TES in dysphagia have been reported in the literature. Information on the effects of stimulation on swallowing physiology is limited (Barikroo et al., 2017; Barikroo et al., 2018; Berretin‐Felix et al., 2016; Carnaby & Harenberg, 2013; Chen et al., 2015; Crary & Carnaby, 2014; Ortega et al., 2016; Park et al., 2009). According to the literature, many questions remain regarding the definition of TES application for dysphagia intervention in adults. It seems that a “one size fits all approach” may be inappropriate. Recent studies controlled scientific rigor, but variability and limited scientific control remain. As such, patient selection, electrode placement, stimulation parameters and exercise programs must be better described (Barikroo et al., 2018; Crary & Carnaby, 2014).

Although the results are encouraging, many aspects require further investigation. The evidence presented is not sufficient to establish TENS as an effective therapeutic approach for speech therapy in cases of voice and swallowing function rehabilitation/habilitation in patients without other associated disorders. It was also not possible to identify technique application patterns, especially in cases of swallowing function. The following are the most limiting factors regarding the level of evidence: the number of study participants; lack of information for randomization; lack of standardized tests for outcomes; and lack of stimulation parameters. More studies are needed to determine standardized application methods and levels of effectiveness.

## 5 CONCLUSION

The following conclusions can be drawn from this review:

- The studies analyzed exhibited medium methodological quality, showing difficulty primarily in the anonymous allocation of participants.
- Different TENS parameters are used. The frequency of the stimuli differed for swallowing functions.
- There are different electrode placements within and between the speech therapy areas.
- In relation to voice, the clinical therapy for dysphonia rehabilitation was better described than the techniques used for dysphagia.
- The voice specialty has more parameters to consider during assessment, some measured using complementary examinations, which seems to better explain the effects of TENS.
- In the area of swallowing function there are many non‐standardized assessment instruments to classify the dysphagia scale.
- There are variations in the time, number and periodicity of the therapeutic sessions for voice and swallowing function.
- There were no statistically significant differences between the use of TENS alone or associated with another therapeutic technique for voice. For swallowing function, one study proved better results in cases of associated techniques – TENS + traditional therapy.
- TENS is not the best strategy to analyze the diadochokinetic parameters of voice.
- The use of TENS on voice seems to demonstrate better effects on vocal quality; influence the comfort and stability of vocal emissions; help in muscle relaxation; and change some of the acoustic voice parameters.
- The use of TES in swallowing functions can target sensitivity or motor muscle responses (contraction or relaxation).
- The use of TENS on swallowing seems to improve swallowing function and pressure, and impact sensitivity to coughing and nutritional status.
- The fact that the studies were heterogeneous made it difficult to determine effectiveness and the parameters to use in future research. Speech therapy should increase the number of studies and improve their methodological quality, reassessing their methodological criteria. Current clinical practice is not grounded on evidence‐based science.

## CONFLICT OF INTEREST

The authors declare that there is no conflict of interest.

## AUTHORS CONTRIBUTIONS

1. Study conception and design: Milena Assis da Silva and Laura Davison Mangilli Toni. 2. Data collection, analysis and interpretation: Milena Assis da Silva and Laura Davison Mangilli Toni. 3. Drafting the article or revising it critically: Milena Assis da Silva and Laura Davison Mangilli Toni. 4. Final approval of the version submitted for publication: Laura Davison Mangilli Toni.

## Data Availability

Data sharing not applicable to this article as no datasets were generated or analysed during the current study

## References

[cre2470-bib-0001] Barikroo, A. , Berretin‐Félix, G. , Carnaby, G. , & Crary, M. (2017). Effect of transcutaneous electrical stimulation amplitude on timing of swallow pressure peaks between healthy young and older adults. Gerodontology, 34, 24–32. 26694095 10.1111/ger.12221

[cre2470-bib-0002] Barikroo, A. , Carnaby, G. , Bolser, D. , Rozensky, R. , & Crary, M. (2018). Transcutaneous electrical stimulation on the anterior neck region: The impact of pulse duration and frequency on maximum amplitude tolerance and perceived discomfort. Journal of Oral Rehabilitation, 45, 436–441. 29574920 10.1111/joor.12625 PMC6206848

[cre2470-bib-0003] Berretin‐Felix, G. , Sia, I. , Barikroo, A. , Carnaby, G. D. , & Crary, M. A. (2016). Immediate effects of transcutaneous electrical stimulation on physiological swallowing effort in older versus young adults. Gerodontology, 33, 348–355. 25393704 10.1111/ger.12166

[cre2470-bib-0004] Bhatt, A. D. , Goodwin, N. , Cash, E. , Bhatt, G. , Silverman, C. L. , Spanos, W. J. , Bumpous, J. M. , Potts, K. , Redman, R. , Allison, W. A. , & Dunlap, N. E. (2015). Impact of transcutaneous neuromuscular electrical stimulation on dysphagia in patients with head and neck cancer treated with definitive chemoradiation. Head & Neck, 37, 1051–1056. 24710791 10.1002/hed.23708

[cre2470-bib-0005] Blumenfeld, L. , Hahn, Y. , LePage, A. , Leonard, R. , & Belafsky, P. C. (2006). Transcutaneous electrical stimulation versus traditional dysphagia therapy: A nonconcurrent cohort study. Otolaryngology–Head and Neck Surgery, 135(5), 754–757. 17071307 10.1016/j.otohns.2006.04.016

[cre2470-bib-0006] Carnaby, G. D. , & Harenberg, L. (2013). What is “usual care” in dysphagia rehabilitation: A survey of USA dysphagia practice patterns. Dysphagia, 28, 567–574. 23670700 10.1007/s00455-013-9467-8

[cre2470-bib-0007] Carnaby‐Mann, G. D. , & Crary, M. A. (2007). Examining the evidence on neuromuscular electrical stimulation for swallowing: A meta‐analysis. Archives of Otolaryngology—Head & Neck Surgery, 133, 564–571. 17576907 10.1001/archotol.133.6.564

[cre2470-bib-0008] Carnaby‐Mann, G. D. , & Crary, M. A. (2008). Adjunctive neuromuscular electrical stimulation for treatment‐refractory dysphagia. The Annals of Otology, Rhinology, and Laryngology, 117, 279–287. 10.1177/000348940811700407 18478837

[cre2470-bib-0009] Chen, Y. W. , Chang, K. H. , Chen, H. C. , Liang, W. M. , Wang, Y. H. , & Lin, Y. N. (2015). The effects of surface neuromuscular electrical stimulation on poststroke dysphagia: A systemic review and meta‐analysis. Clinical Rehabilitation, 30, 24–35. 25697453 10.1177/0269215515571681

[cre2470-bib-0010] Conde, M. C. M. , Siqueira, L. T. , Vendramini, J. E. , Brasolotto, A. G. , Guirro, R. R. J. , & Silvério, K. C. A. (2017). Transcutaneous electrical nerve stimulation (TENS) and laryngeal manual therapy (LMT): Immediate effects in women with dysphonia. Journal of Voice, 32, 385.e17–385.e25. 10.1016/j.jvoice.2017.04.019 28533075

[cre2470-bib-0011] Crary, M. A. , & Carnaby, G. D. (2014). Adoption into clinical practice of two therapies to manage swallowing disorders: Exercise‐based swallowing rehabilitation and electrical stimulation. Current Opinion in Otolaryngology & Head and Neck Surgery, 22, 172–180. 24675153 10.1097/MOO.0000000000000055 PMC4104745

[cre2470-bib-0012] Doucet, B. M. , Lam, A. , & Griffin, L. (2012). Neuromuscular electrical stimulation for skeletal muscle functions. The Yale Journal of Biology and Medicine, 85, 201–215. 22737049 PMC3375668

[cre2470-bib-0013] Fabron, E. M. G. , Petrini, A. S. , Cardoso, V. M. , Batista, J. C. T. , Motonaga, S. M. , & Marino, V. C. C. (2017). Immediate effects of tongue trills associated with transcutaneous electrical nerve stimulation (TENS). CoDAS, 29(3), e20150311. 28614457 10.1590/2317-1782/20172015311

[cre2470-bib-0014] Fowler, L. P. , Gorham‐Rowan, M. , & Hapner, E. R. (2011). An exploratory study of voice change associated with healthy speakers after transcutaneous electrical stimulation to laryngeal muscles. Journal of Voice, 25, 54–61. 20080024 10.1016/j.jvoice.2009.07.006

[cre2470-bib-0015] Glanz, M. , Klawansky, S. , Stason, W. , Berkey, C. , & Chalmers, T. C. (1996). Functional electrostimulation in poststroke rehabilitation: A meta‐analysis of randomized controlled trials. Archives of Physical Medicine and Rehabilitation, 77, 549–553. 8831470 10.1016/s0003-9993(96)90293-2

[cre2470-bib-0016] Guirro, R. R. J. , Bigaton, D. R. , Silvério, K. C. A. , Berni, K. C. S. , Distéfano, G. , Santos, F. L. , & Forti, F. (2008). Transcutaneous electrical nerve stimulation in dysphonic women. Pró‐Fono Revista de Atualização Científica, 20(3), 189–194. 18852967 10.1590/s0104-56872008000300009

[cre2470-bib-0017] Higgins, J. P. T. , Thomas, J. , Chandler, J. , Cumpston, M. , Li, T. , Page, M. J. , Welch, V. A. (Eds.). Cochrane handbook for systematic reviews of interventions. 2nd Edition. Chichester, UK: John Wiley & Sons; 2019.

[cre2470-bib-0018] Humbert, I. A. , Michou, E. , MacRae, P. R. , & Crujido, L. (2012). Electrical stimulation and swallowing: How much do we know? Seminars in Speech and Language, 33, 203–216. 22851342 10.1055/s-0032-1320040 PMC3475497

[cre2470-bib-0019] Kitchen, S. (2001). Electrotherapy ‐ evidence‐based practice (11th ed.). Churchill Livingstone.

[cre2470-bib-0020] Komi, P. (1986). Training of muscle strength and power: Interaction of neuromotoric, hypertrophic, and mechanical factors. International Journal of Sports Medicine, 7(S1), S10–S15. 10.1055/s-2008-1025796 3744642

[cre2470-bib-0021] Lim, K. B. , Lee, H. J. , Lim, S. S. , & Choi, Y. I. (2009). Neuromuscular electrical and thermal‐tactile stimulation for dysphagia caused by stroke: A randomized controlled trial. Journal of Rehabilitation Medicine, 41, 174–178. 19229451 10.2340/16501977-0317

[cre2470-bib-0022] Maeda, K. , Koga, T. , & Akagi, J. (2017). Interferential current sensory stimulation, through the neck skin, improves airway defense and oral nutrition intake in patients with dysphagia: A double‐blind randomized controlled trial. Clinical Interventions in Aging, 12, 1879–1886. 29158670 10.2147/CIA.S140746 PMC5683771

[cre2470-bib-0023] Mansuri, B. , Torabinejhad, F. , Jamshidi, A. A. , Dabirmoghaddam, P. , Vasaghi‐Gharamaleki, B. , & Ghelichi, L. (2020). Application of high‐frequency transcutaneous eletrical nerve stimulation in muscle tension dysphonia patiets with the pain complaint: The immediate effect. Journal of Voice, 34(5), 657–666. 31078355 10.1016/j.jvoice.2019.02.009

[cre2470-bib-0024] Ortega, O. , Rofes, L. , Martin, A. , Arreda, V. , López, I. , & Clave, P. (2016). A comparative study between two sensory stimulation strategies after two weeks treatment on older patients with Oropharyngeal dysphagia. Dysphagia, 31, 706–716. 27503566 10.1007/s00455-016-9736-4

[cre2470-bib-0025] Park, J. W. , KIm, Y. , Oh, J. C. , & Lee, H. J. (2012). Effortful swallowing training combined with electrical stimulation in poststroke dysphagia: A randomized controlled study. Dysphagia, 27, 521–527. 22447240 10.1007/s00455-012-9403-3

[cre2470-bib-0026] Park, J. W. , Oh, J. C. , Lee, H. J. , Park, S. J. , Yoon, T. S. , & Kwon, B. S. (2009). Effortful swallowing training coupled with electrical stimulation leads to an increase in hyoid elevation during swallowing. Dysphagia, 24, 296–301. 19255707 10.1007/s00455-008-9205-9

[cre2470-bib-0027] Rofes, L. , Cola, P. , & Clave, P. (2014). The effects of sensory stimulation on neurogenic oropharyngeal dysphagia. Journal of Gastroenterology and Hepatology Research, 3, 1066–1072.

[cre2470-bib-0028] Ryu, J. S. , Kang, J. Y. , Park, J. Y. , Nam, S. Y. , Choi, S. H. , Roh, J. L. , Kim, S. Y. , & Choi, K. H. (2009). The effect of electrical stimulation therapy on dysphagia following treatment for head and neck cancer. Oral Oncology, 45, 665–668. 19095492 10.1016/j.oraloncology.2008.10.005

[cre2470-bib-0029] Sampaio, R. , & Mancini, M. (2017). Systematic review studies: A guide for careful synthesis of the scientific evidence. Brazilian Journal of Physical Therapy, 11, 83–89.

[cre2470-bib-0030] Santos, J. K. O. , Silvério, K. C. A. , Oliveira, N. F. C. D. , & Gama, A. C. C. (2016). Evaluation of electrostimulation effect in women with vocal nodules. Journal of Voice, 30, 769.e1–769.e7. 10.1016/j.jvoice.2015.10.023 26822388

[cre2470-bib-0031] Shiwa, S. R. , Costa, L. O. P. , Moser, A. D. L. , Aguiar, I. C. , & Oliveira, L. V. F. (2011). PEDro: The physiotherapy evidence database. Fisioterapia em Movimento, 24, 523–533.

[cre2470-bib-0032] Silvério, K. C. A. , Brasolotto, A. G. , Siqueira, L. T. D. , Carneiro, C. G. , Fukushiro, A. P. , & Guirro, R. R. J. (2015). Effect of application of transcutaneous electrical nerve stimulation and laryngeal manual therapy in dysphonic women: Clinical trial. Journal of Voice, 29, 200–208. 25439510 10.1016/j.jvoice.2014.06.003

[cre2470-bib-0033] Simonelli, M. , Ruoppolo, G. , Iosa, M. , Morone, G. , Fusco, A. , Grasso, M. G. , Gallo, A. , & Paolucci, S. (2019). A stimulus for eating. The use of neuromuscular transcutaneous electrical stimulation in patients affected by severe dysphagia after subacute stroke: A pilot randomized controlled trial. NeuroRehabilitation, 44, 103–110. 30714980 10.3233/NRE-182526

[cre2470-bib-0034] Siqueira, L. T. D. , Silvério, K. C. A. , Brasolotto, A. G. , Guirro, R. R. J. , Carneiro, C. G. , & Behlau, M. (2017). Effects of laryngeal manual therapy (LMT) and transcutaneous electrical nerve stimulation (TENS) in vocal folds diadochokinesis of dysphonic women: A randomized clinical trial. CoDAS, 29, e20160191. 28538831 10.1590/2317-1782/20172016191

[cre2470-bib-0035] Sluka, K. A. , & Walsh, D. M. (2003). Transcutaneous electrical nerve stimulation: Basic science mechanisms and clinical effectiveness. The Journal of Pain, 4, 109–121. 14622708 10.1054/jpai.2003.434

[cre2470-bib-0036] Stangherlin, D. A. C. , Lemos, I. O. , Bello, J. Z. , & Cassol, M. (2020). Transcutaneous electrical nerve stimulation in dysphonic patients: A systematic review. Journal of Voice, S0892‐1997(20)30091‐6. 10.1016/j.jvoice.2020.03.003. 32273210

[cre2470-bib-0037] Sun, S. F. , Hsu, C. W. , Lin, H. S. , Sun, H. P. , Chang, P. H. , Hsieh, W. L. , & Wnag, J. L. (2013). Combined neuromuscular electrical stimulation (NMES) with fiberoptic endoscopic evaluation of swallowing (FEES) and traditional swallowing rehabilitation in the treatment of stroke‐related. Dysphagia, 28, 557–566. 23584790 10.1007/s00455-013-9466-9

